# Supplementary figures and images for: Increased signal diversity/complexity of spontaneous EEG, but not evoked EEG responses, in ketamine-induced psychedelic state in humans
Source: PLoS One. 2020 Nov 23;15(11):e0242056. doi: 10.1371/journal.pone.0242056 (PMC7682856; doi:10.1371/journal.pone.0242056)

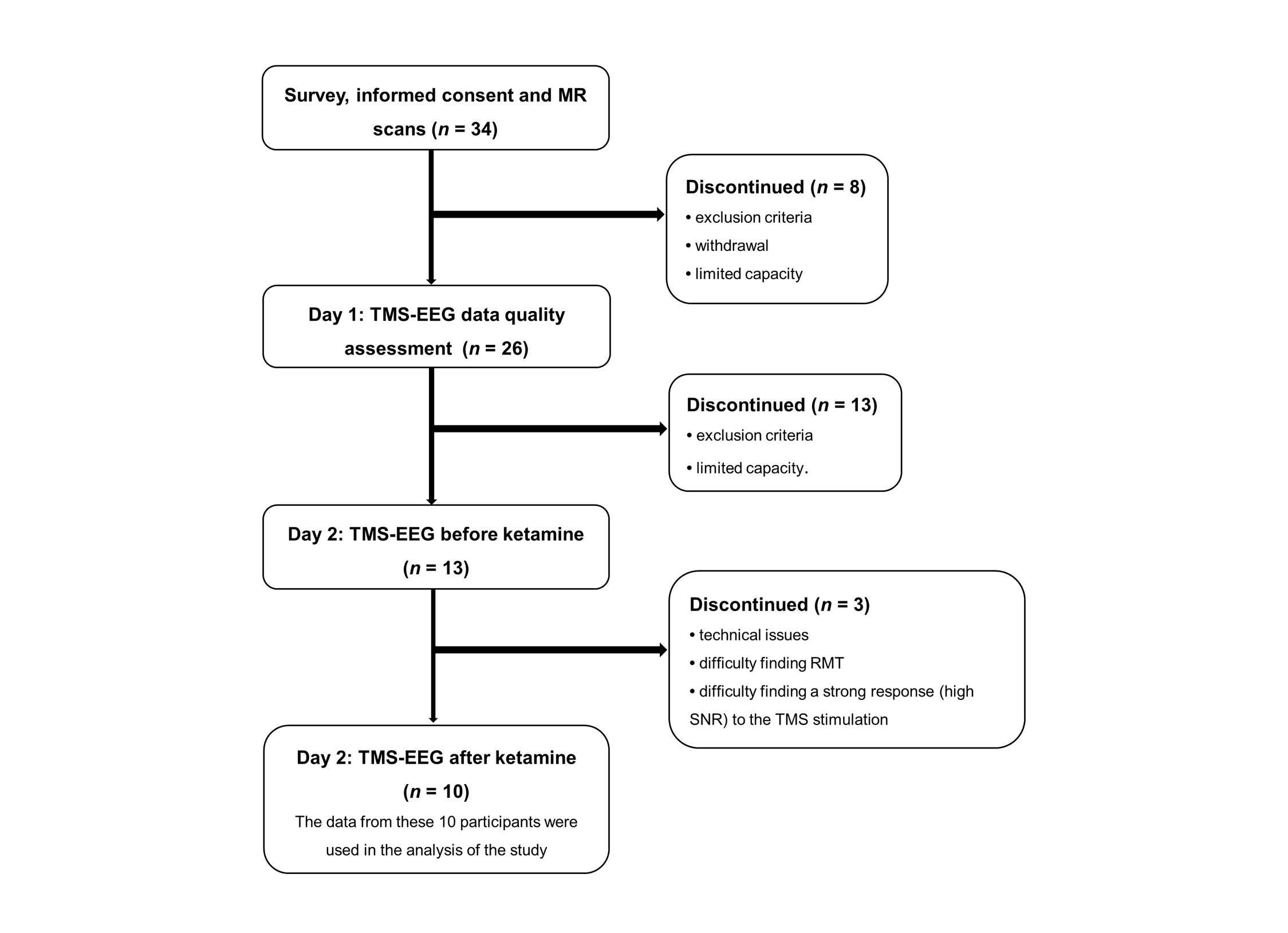

Supplement: S1 Fig — The number of participants included (left) or discontinued (right) at each stage of the study is indicated with n. In the end 10 participants completed the study. (TIF) [file pone.0242056.s001.tif]

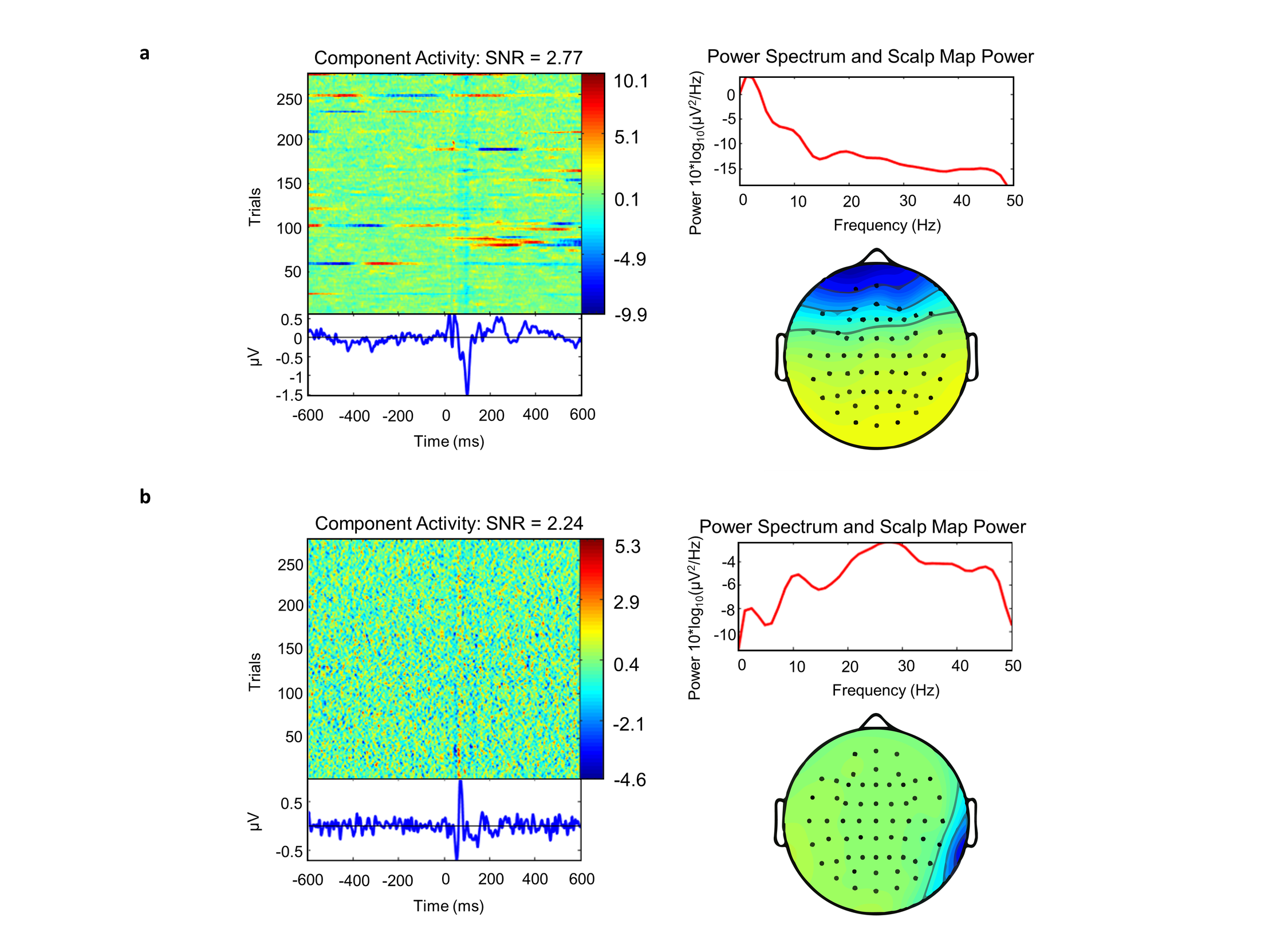

Supplement: S2 Fig — Example of (a) eyeblink artefact and (b) muscle artefact. The left panel shows component activity for all trials (above) and the average of all trials (below) and to the right. The right panel shows power spectrum and scalp map power for the components. (TIF) [file pone.0242056.s002.tif]

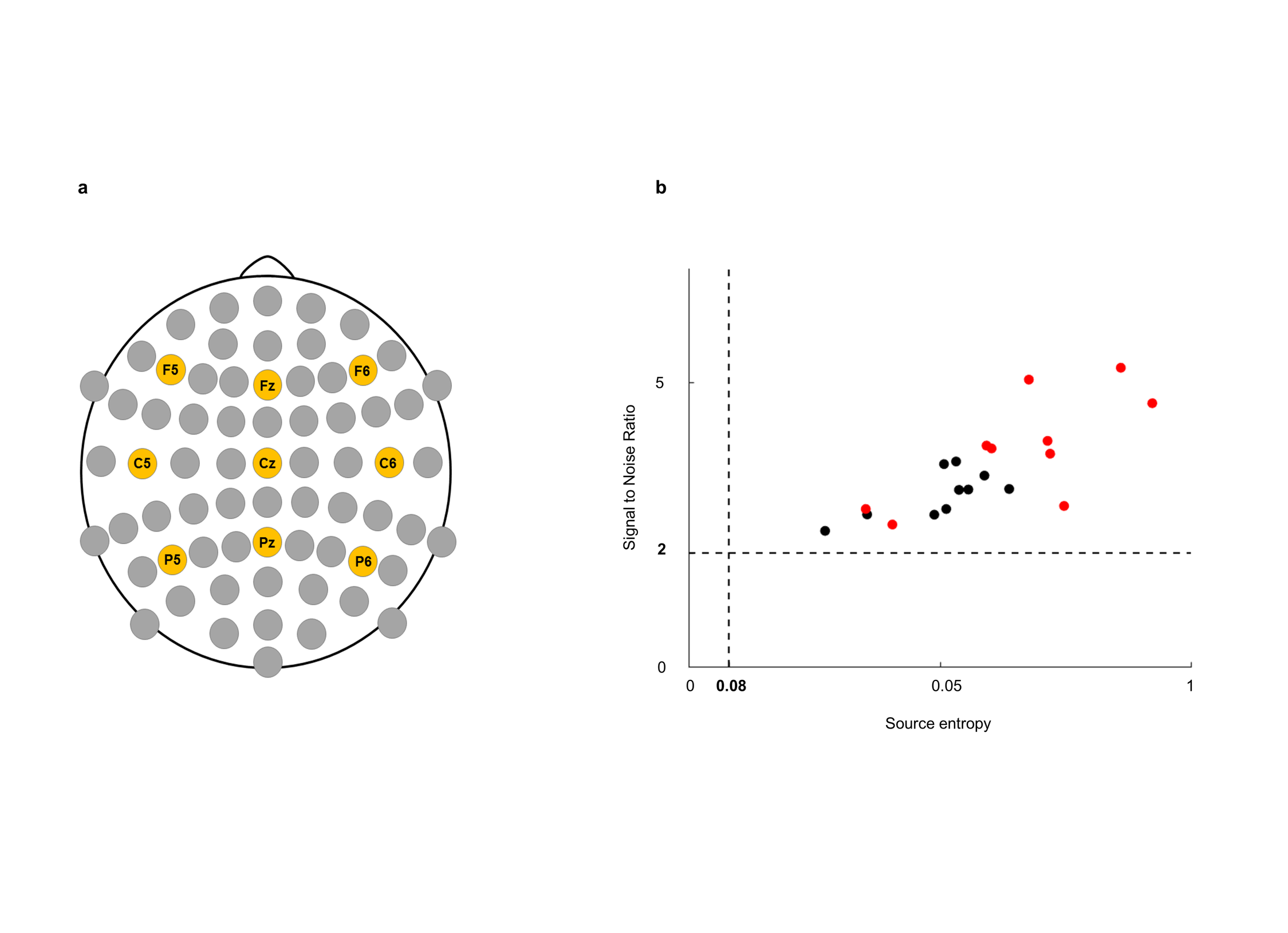

Supplement: S3 Fig — (a) Spontaneous signal diversity was calculated from 9 channels (yellow) distributed across the scalp: F5, Fz, F6, C6, Cz, C6, P5, Pz, and P6. (b) Signal-to-noise ratio (SNR) and source entropy (H) for all TMS-EEG measurements and participants. The black dots are participants during normal wakefulness and the red dots are participants during sub-anaesthetic ketamine. The source entropy of the data was above 0.08 and all data showed a high SNR (> 2). None of the data were excluded based on too low source entropy. (TIF) [file pone.0242056.s003.tif]

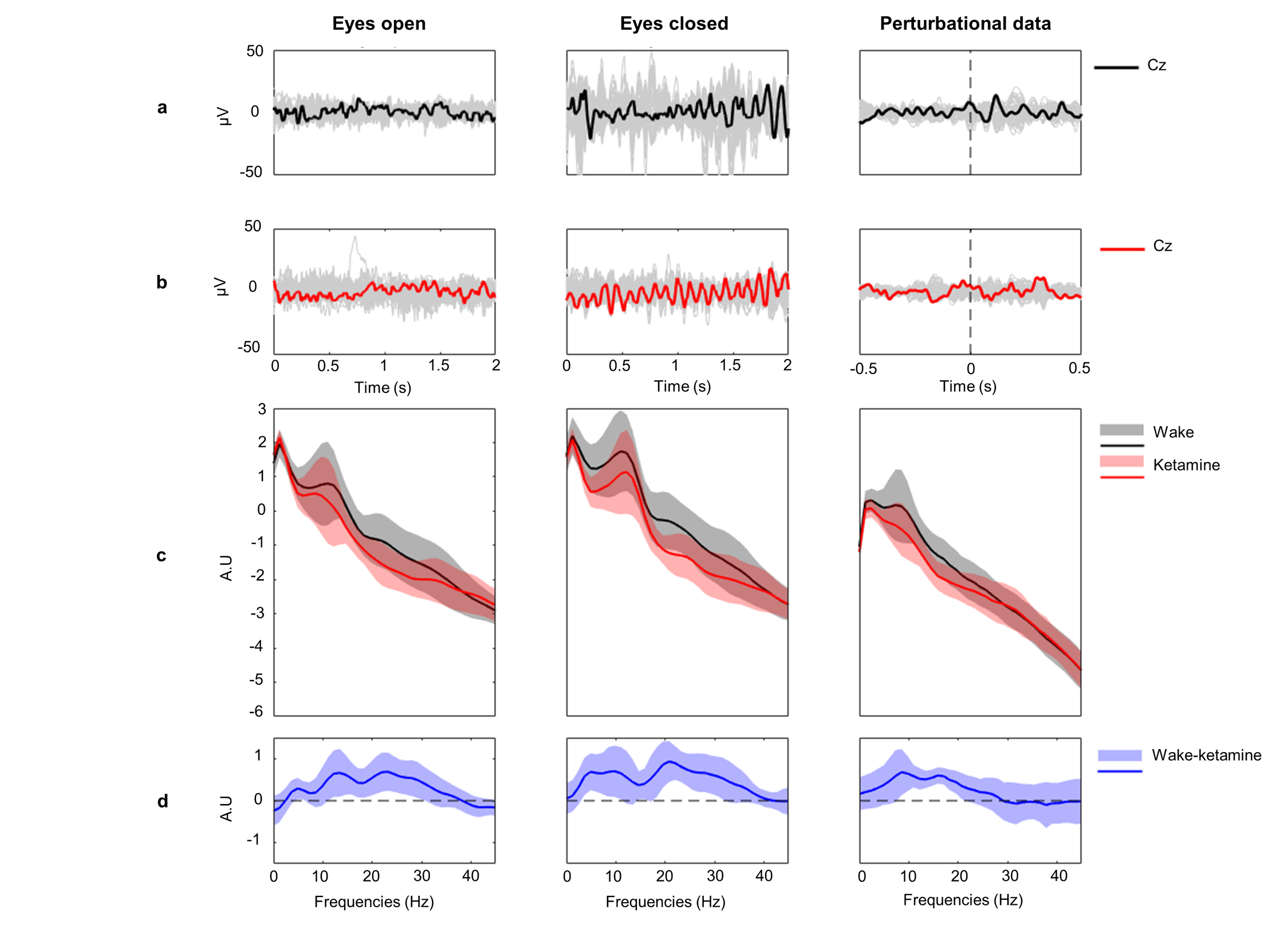

Supplement: S4 Fig — Here we show examples of processed data from within a single epoch in the awake (a) and ketamine (b) conditions, as well as mean power spectral densities (PSD) across participants for wakefulness and ketamine (c) and the within subject, pairwise mean difference between wake and ketamine PSDs (d). Each of these are shown for the recordings of spontaneous activity in the resting state with eyes open (column 1) and resting state with eyes closed (column 2), as well as in response to TMS stimulation with eyes open (third column). In (a) and (b), the thick line shows the activity of channel Cz in the single epoch, while the gray lines in the background shows the activity of the remaining channels in that same epoch. For column 3, the black dashed line shows the time of the TMS pulse. In (c), the PSD is shown between 0 and 45 Hz, for the wakefulness (black) and ketamine (red) conditions. The thick line indicates the mean across participants, while the shaded area shows the extent of 3 standard errors of the mean. For the third column, the PSD is calculated only for the data after the pulse (0-500ms). The thick blue line in (d) shows the mean pairwise difference in the PSD (within participants) while the shaded area again shows the extent of 3 standard errors of the mean. Here we see that the frequency bands changes in the ketamine versus wakefulness states differ between the data types: in the eyes open condition, differences appear in the beta and low gamma bands (~12-30Hz); in the eyes closed condition, differences appear in theta, alpha, and low gamma bands (~4–12 Hz and ~20–30 Hz); in the perturbed data, in the alpha and beta bands (~8–20 Hz). (TIF) [file pone.0242056.s004.tif]

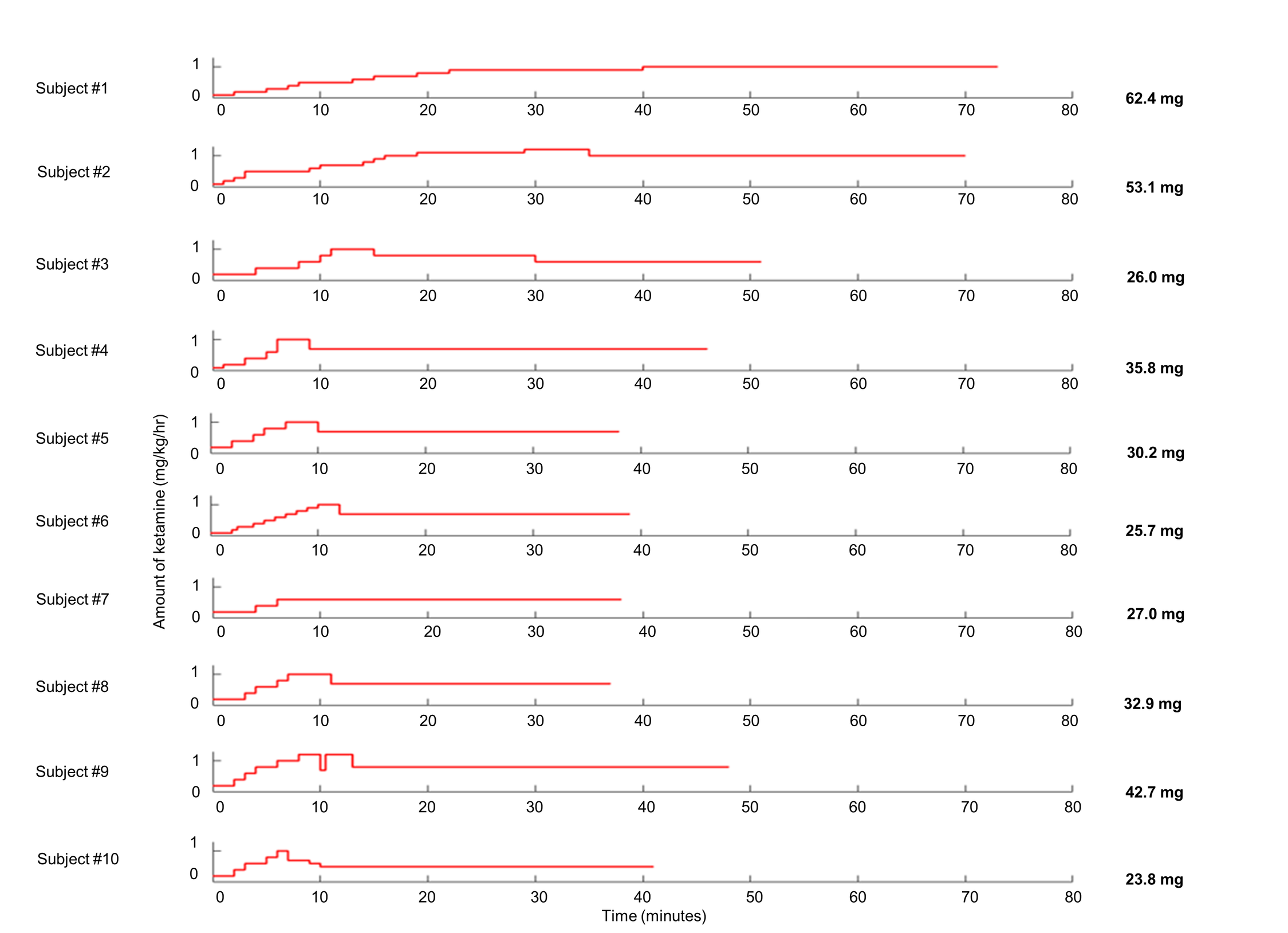

Supplement: S5 Fig — The plots show the time course of ketamine infusion rates, between 0.1 and 1.2 mg/kg/hr for the ten subjects. The infusion was eventually stabilized (0.1–1.0 mg/kg/hr) at a constant rate in the final period, during which, all the TMS-EEG data used in the study were collected. The total amount of ketamine given is shown to the right. (TIF) [file pone.0242056.s005.tif]

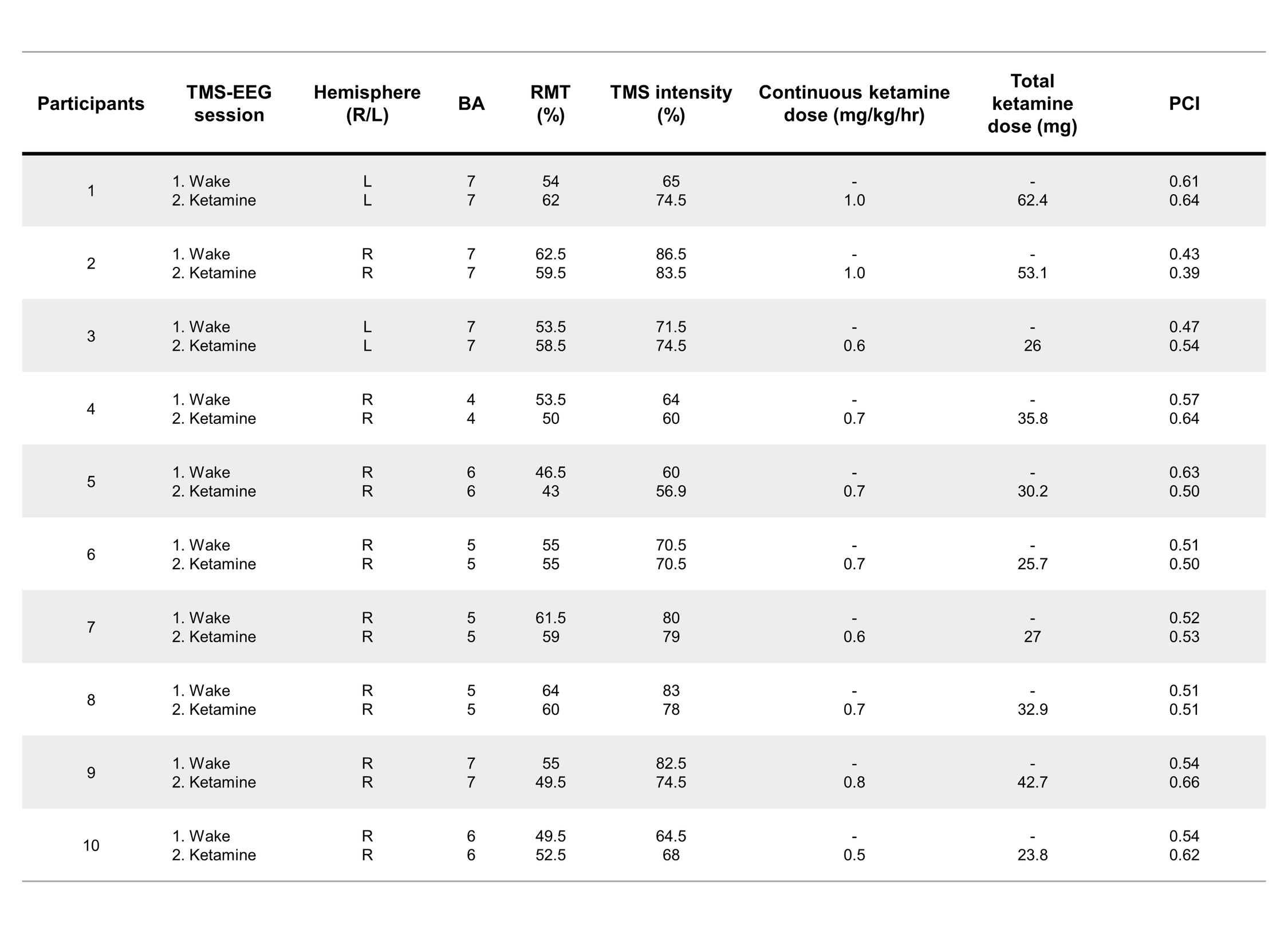

Supplement: S1 Table — PCI values in bold were used in the present study. R = right, L = left, BA = Brodmann Area, % = percentage of maximal stimulator output. (TIF) [file pone.0242056.s006.tif]
