# Supplementary material for: Increased signal diversity/complexity of spontaneous EEG, but not evoked EEG responses, in ketamine-induced psychedelic state in humans
Source: PLoS One. 2020 Nov 23;15(11):e0242056. doi: 10.1371/journal.pone.0242056 (PMC7682856; doi:10.1371/journal.pone.0242056)
Supplement: Attachment — Submitted filename: Response_to_reviewers_PLOS_ONE.docx [file pone.0242056.s007.docx]

Dear Editor,

Thank you for the opportunity to improve our manuscript for publication.This document contains our responses to the reviewer comments. We have marked all changes in the updated manuscript with yellow highlight. Our responses are given below the comments and are coloured green and manuscript revisions that are directly relevant to the reviewer comments or our responses to them are coloured with a yellow highlight in Calibri font. Relevant segments from the original manuscript that supplement our responses to the reviewers are in black Calibri font.

We hope our revisions to the manuscript and responses to the reviewers’ comments will be sufficient for it to be published in PLOS ONE.

Sincerely,

N. Farnes, B.E. Juel, A.S. Nilsen, L. Romundstad, and J.F. Storm

_________________________________________________________________________

**Reviewer #1**: The authors present a study showing changes in spontaneous electroencephalogram (EEG) signals - increased signal diversity/complexity - but not in evoked EEG responses in ketamine-induced psychedelic state in humans. The work is novel and the experiments seem to have been conducted rigorously. However, there are some issues with the manuscript that need to be addressed, and things that need to be clarified and explained in greater detail.

Thank you for detailed feedback. We are glad you find our work novel with rigorously conducted experiments and we hope our clarification to your comments suffice for your approval of the manuscript for publication.

**Major points:**

- The authors mention several times the term "signal diversity" but fail to describe it from a mathematical point of view. What makes a signal diverse? Furthermore, how can "signal diversity" be quantified? Is diversity used in the same sense as complexity? If that is the case, what definition of complexity is being used by the authors? The review paper by Tononi et al. (1998) presents different kinds of complexity. Does "signal diversity" correspond to one of those types of complexity?

- Thank you for this comment. We agree that the use of this terminology is not entirely clear and have added some clarifications to the manuscript. For us, signal diversity is a general term for measures which quantify the variation of neural signals in a given physiological state, and not strictly defined by a single mathematical formula. In the manuscript it is used for measures which quantify differentiation in the signal alone (using measures that are maximal when the binary time series are completely noisy: LZc, ACE and SCE), while we reserve the term “complexity measures” for measures (like PCI) that simultaneously capture both integration and differentiation as per Oizumi (2014) and Tononi (1998, the term neural differentiation in the manuscript is analogous with the term information used in the article).
- Although we consider this to be covered in the manuscript, we have have made the following changes to the manuscript for clarification:
  - While PCI aims to capture both neural differentiation and integration, inspired by IIT ( Casali et al. 2013), recent studies have indicated that measures solely capturing differentiation in recordings of spontaneous brain activity, seem to vary with brain state in parallel with PCI [10,16]. Here, we label such measures of differentiation as “signal diversity measures” in line with Shartner et al. (2017) . Thus, a signal diversity measure quantifies the variation of neural signals in a given physiological brain state and provides an estimate of the differentiation in the signal, although generally accepted, sharp, mathematical definition of this concept seems to be lacking.

- Significant differences (figure 2) are reported using Lempel-Ziv complexity (LZc), with authors claiming that these reflect changes to "signal diversity". Have the authors checked the spectra of the signals and evaluated any possible differences using linear methods based on the Fourier transform? In some cases LZc might be only reflecting changes that can be observed with spectral methods. I would ask the authors to use surrogate data analysis to test the validity of their results in the context of complexity changes.

- We have not looked into changes to the frequency spectra using linear methods based on the Fourier transform, as it was outside the scope of the study. It is indeed likely that the spectra change reliably between the wake and ketamine conditions, and that those changes might even be used to explain the observed differences in the signal diversity. That said, even if the differences in signal diversity could be completely predicted by changes in power spectra, we would argue that this would not invalidate the claim that the signal diversity has changed. In other words, even if surrogate data (generated with power spectra identical to those found in the different conditions) would show that the signal diversity differences observed in the original data were not bigger than those generated by the surrogate process, we feel that this would not invalidate the claims made in this paper. While we are not trained in surrogate data analysis, and are therefore weary of adding such an analysis in the last minute, we do include a new figure in the supporting information illustrating spectral changes in the different conditions:
  - **S4 Fig. Spectral characteristics of the data.** Here we show examples of processed data from within a single epoch in the awake (a) and ketamine (b) conditions, as well as mean power spectral densities (PSD) across participants for wakefulness and ketamine (c) and the within subject, pairwise mean difference between wake and ketamine PSDs (d). Each of these are shown for the recordings of spontaneous activity in the resting state with eyes open (column 1) and resting state with eyes closed (column 2), as well as in response to TMS stimulation with eyes open (third column). In (a) and (b), the thick line shows the activity of channel Cz in the single epoch, while the gray lines in the background shows the activity of the remaining channels in that same epoch. For column 3, the black dashed line shows the time of the TMS pulse. In (c), the PSD is shown between 0 and 45 Hz, for the wakefulness (black) and ketamine (red) conditions. The thick line indicates the mean across participants, while the shaded area shows the extent of 3 standard errors of the mean. For the third column, the PSD is calculated only for the data after the pulse (0-500ms). The thick blue line in (d) shows the mean pairwise difference in the PSD (within participants) while the shaded area again shows the extent of 3 standard errors of the mean. Here we see that the frequency bands changes in the ketamine versus wakefulness states differ between the data types: in the eyes open condition, differences appear in the beta and low gamma bands (~12-30Hz); in the eyes closed condition, differences appear in theta, alpha, and low gamma bands (~4-12 Hz and ~20-30 Hz); in the perturbed data, in the alpha and beta bands (~8-20 Hz).

We also point to these analyses in the discussion:

- Spectral analyses show that power spectrum density differences between wake and ketamine change for spontaneous data with eyes open and eyes closed, as well as for perturbational data, with different frequency band changes for each condition (S4 Fig). This is in line with findings of a lack of visual input increases synchronous alpha band activity [40]
- Although perturbational data show frequency band changes in wake versus ketamine data, PCI seems less sensitive to changes in content (e.g. induced by removing visual aspects from phenomenal experience) within a global physiological state (here: being fully awake).

- The authors claim that "PCI is a measure of the global, spatio-temporal complexity of evoked, cortical responses to a local perturbation". Given that EEG signals are heavily affected by volume conduction, would this affect PCI results? This should be discussed.

- While the reviewer is correct that scalp EEG signals are heavily affected by volume conduction, it is unlikely to be an issue for PCI given the current implementation. If using raw EEG signals to calculate PCI, the complexity of patterns might be misrepresented, for example because different signals from distinct areas may appear linked even though they are not. Furthermore, even if volume conduction is an issue for the precise estimation of the complexity of the brain, we see no good reason why it would be differently affecting the signals in the wakeful vs ketamine conditions. Thus, it should not bias the results here, and only serve to cause the PCI to underestimate the true brain complexity due to the spatial activity appearing more synchronous than it is in reality.

- Line 114: The authors mention that LZc, amplitude coalition entropy (ACE), and synchrony coalition entropy (SCE) capture how "disordered" a signal is. Is this really the case? Do LZc, ACE and SCE return the highest values when analysing signals showing complete disorder (i.e. noise)? This needs to be investigated and discussed.

- Yes. Feeding a completely random binary signal to any of the algorithms will yield the maximal possible values for the measures (in the limit of infinitely long signals). This is part of the reason why we opt for normalizing the raw measures against the values of the measures calculated from the shuffled signals. This is one of the issues with observational measures (compared to perturbationally based ones): they cannot distinguish signals generated from very complex architectures from those that are generated by noise processes. We have taken this to account and amended the discussion part of the manuscript:
  - One aspect that strongly distinguishes signal diversity measures from PCI is that the signal diversity measures will increase when processes in the brain grow increasingly noisy or complex, while PCI will only increase if the underlying brain processes get more complex. This is largely an issue related to the fact that signal diversity measures are dependent on observational data, while the PCI is calculated from controlled perturbations and subsequent observations of the brain. Thus, conditions where signal diversity measures increase, but PCI remains unchanged may be an indicator that the brain state is more chaotic, but not more complex.

- Line 156: The aim of the study is stated, but what was the research hypothesis?

- Thank you for the comment. You are correct that our research hypothesis is not clearly stated. This has now been changed by the addition of the following sentence to the introduction:
  - Given that previous results from the literature regarding signal diversity replicate, and an assumption that PCI and signal diversity measures covary in healthy humans, we hypothesized that PCI would increase in the psychedelic state compared to normal wakefulness.

- Line 326: How many data samples were available for the calculation of LZc? Have the authors checked that the number of data points is enough for a stable and reliable estimate of the LZc of such a short signal?

- This is an important point to consider, and changing the parameters used in the analysis will definitely change the numbers that result. Therefore, we should be careful with too strong comparisons of values between studies unless the parameters for the analysis were the same. For example, changing the length of the signal may change the absolute values obtained by signal diversity estimates. One reason for this is that the longer the signal gets, the more likely it is that a shuffled version of the signal will actually be a maximally complex signal (the higher the N, the higher the proportion of strings of length N are maximally complex). However, as long as the length of the signals are kept stable between conditions, the comparisons should be valid. The more important aspect to worry about though, is an issue of ceiling effects - for short signals,
- Each epoch in our data contained 18000 samples per epoch, which yields very stable estimates of the LZc of EEG signals in these conditions. The constraints were more serious for ACE and SCE, which require estimation of a distribution, where one sample requires a value from each channel (to define the “coalition” at that point). We discussed it at length in the methods section:
  - “From the 62 channels, only 9 were chosen for signal diversity analysis (Fig S32) due to the entropy measures being calculated based on the distribution of states observed in the data. Since number of states, S, available in a network of N binary nodes is S = 2N, and the measures require an estimate of the probability density distribution over states, the number of samples in an epoch should be at least as large as the number of states available to yield a representative estimate of the underlying distribution of states. With each epoch containing 2000 samples (8s epoch length x 250Hz sampling rate), the maximal number of channels that could be included in the analysis if all states were to have a chance of being sampled at least once, would be 10. However, to increase the chance of getting a decent sampling of the distribution, we decided to use only 9 channels.”

- Line 359: Why was the mean and not the median used as the threshold for the calculation of LZc and ACE? The median is much more robust to any outliers in the data. I would recommend comparing the results obtained with the mean to those that would be obtained using the median instead.

- You are correct, the median is the more robust estimator of the distribution mode and could arguably have been used both for estimating thresholds and summarizing diversity across trials. That said, we wanted to use similar thresholds as Schartner et al. (2015), being able to compare our results with their findings.

**Minor points:**

- Line 78: Please give some details on the recent discoveries mentioned in this sentence.

- We have elaborated the sentence to include examples:
  - ..and have been crucial for a series of recent discoveries in brain function. These include changes in network activity and connectivity in the anaesthetized state compared to the normal wakeful state using non-invasive neural measures such as fMRI, TMS and EEG [7,10].

- Line 184: Please include details about the age and sex of the participants in the study. EEG complexity changes with age and/or sex.

- We understand that the reviewer is calling for descriptive information as well as how the complexity measures change with age and sex. We have included the descriptives in the method section of the manuscript:
  - (7 men and 3 women, median age 27.5, range: 21 – 44 years old).
- As for analyses on how complexity changes with age and sex, the small sample size of the study limits the statistical power to find such differences. We have included this in the limitation section of the discussion:
  - A small sample size is associated with higher variability and lower reliability of statistical analyses, for example of correlation, also limiting the investigation of interaction effects of variables such as gender and age. Future studies might thus benefit from a larger sample size.

- Line 299: Who was responsible for the visual inspection of the signals and selection of those for further analysis? A trained expert?

- Yes, a trained analyst was responsible for the visual inspection:
  - EEG responses to TMS were visually inspected by a trained analyst to identify artefactual trials and channels containing abnormal amplitude activity which were excluded from further analysis

- Line 307 to 309: Please include graphical examples of components with artefacts.

- Two graphical examples of components with artefacts have been included in the supporting information
  - **Fig S2. Example of artifactual components.** Example of **a)** eyeblink artefact and **b)** muscle artefact. The left panel shows component activity for all trials (above) and the average of all trials (below). The right panel shows power spectrum and scalp map power for the components.

- Lines 314 and 342: What were the orders of the filters?

- We used default/standard settings for the filter order. In other words, the order was set to 1.5 times the sampling rate (3*srate/2). This is now mentioned in the manuscript:
  - “The filter order was kept to its default value (3*rate/2)”
  - (Hamming window sinc FIR filter, default filter order value (3*rate/2)),

- Line 315: Why were the signals down-sampled to a sampling frequency different to the one used in the spontaneous EEG recordings?

- We used parameters and settings for the PCI analysis based on advice from the team of scientists that developed the PCI measure (Casarotto et al 2016). In fact, as mentioned in the manuscript, we used their software (SiSyPhus Project) and stuck to the parameter choices they had as standard. This was also done in order to avoid compatibility issues related to the methods used for source reconstruction and PCI calculations (also based on scripts from the Massimini group). To clarify this, we added a sentence in the methods section:
  - In general, we aimed to stay as close as possible to the analysis described by the developers of PCI [12-14]. Thus, signals were referenced to the common average reference, using a 1 Hz high pass filter and 45 Hz low pass filter (Hamming window sinc FIR filter) to avoid the need for a notch filter to remove line noise, and downsampled to 312.5 Hz.

- Line 339: Why were artefactual channels interpolated? Explain what you mean by this.

- We interpolated channels in the analysis of both spontaneous and perturbed data. Interpolation of EEG channels is a relatively standard approach in EEG data pre-processing, where the time-series of a channel is replaced by a weighted sum of the recorded activity of other channels. The main reason for doing so to maintain the same number of channels in the data for each participant, while removing the impact of artefactual channels (e.g. through average referencing and spatial filters). In the PCI-analysis, interpolation is important for maintaining the correct channel number for the source reconstruction. In the resting state recordings, interpolation would be important if one of the 9 channels chosen for the signal diversity estimation turned out to be artifactual (which would have left us with fewer channels for some subjects, and might have impacted the results unnecessarily).

- Line 362: What was the rationale for only analysing 9 channels out of 62 instead of 10? The explanation provided is not clear enough.

- Since the entropy measures are calculated from an empirical estimate of the distribution of states (coalitions), we wanted to make sure we obtained a reasonable estimate of the distribution. For every channel included, the number of states (coalitions) available doubles, and the number of data points required to estimate the distribution over state increases proportionally to that. For 9 channels, assuming states being independent, and all states being equally probable, the number of samples required to expect ‘at least one sample in each state’ is ~2100 samples, according to preliminary simulation studies we ran. Thus, even using 9 channels is pushing it given our sampling rate and segment length (and increasing either will make the samples more dependent and make the sample less stationary, respectively). In other words, we think that it is, in fact, the addition of an extra channel that would require an explanation, and not only including 9. Still, we update the text to make our reasoning more obvious to the reader:
  - With each epoch containing 2000 samples (8s epoch length x 250Hz sampling rate), the maximal number of channels that could be included in the analysis if all states were to have a chance of being sampled at least once, would be 10 (# available states = 2^10 = 1024). However, using a simulation study we estimated the number of samples required to expect sampling each state at least once to be ~2100 even with only 9 channels included (results not shown). Thus, to increase the chance of getting a decent sampling of the distribution, even in a condition yielding the maximum entropy distribution of coalition, we decided to use only 9 channels. It should be noted that our estimation assumes that the samples are independent, which is not a good assumption for EEG data, indicating that our inclusion of 9 channels may, if anything, be too optimistic with regards to obtaining a good estimate of the distribution of coalitions.

- Lines 367 and 368: What was the rationale for using the normalisation detailed therein instead of the usual one applied when computing LZc from a signal?

- In addition to the most comparable studies using the shuffling strategy for normalization (e.g. Schartner et al 2017), there are two main reasons for the choice. 1) the shuffling approach gives an estimate of the upper bound of signal diversity for a segment with the same source entropy. Thus, it does not necessarily favor (yield higher estimates for) signals that have a more equal distribution of 0’s and 1’s in their binarized form. 2)

- Lines 517 and 518: How much of it is integration and how much is just merely a volume conduction effect?

- We think it is unlikely that the data underlying PCI are particularly prone to volume conduction effects. If the global response to the local perturbation (TMS) was dominated by volume conduction, the spatial pattern would not be differentiated. Furthermore, the delay (from 10’s to 100’s of milliseconds) between the perturbation and the cortical responses recorded elsewhere on the scalp indicates that the observed activity is not due to volume conduction. In general, the underlying methodology required for the PCI (Massimini et al., 2009). A perturbational approach for evaluating the brain’s capacity for consciousness.) as well as the way the PCI is quantified (Casali et al., 2013). A theoretically based index of consciousness independent of sensory processing and behavior) gives reason to believe that the measure can reasonably be thought to reflect the effective integration in the brain.

- Lines 580 and 581: Does it make sense to evaluate correlation with such a small sample size?

- It is correct that larger sample sizes are more reliable when using correlation analyses. We used correlation because we wanted to compare our results with Schartner et al. (2017). We have updated the discussion to account for this perspective:
  - A small sample size is associated with higher variability and lower reliability of statistical analyses, for example of correlation, also limiting the investigation of interaction effects of variables such as gender and age. Future studies might thus benefit from a larger sample size. However, the sample size used in the current study is larger than in [13] where no difference in PCI was found for anaesthetic doses of ketamine compared to normal wakefulness, and our spontaneous signal diversity results and correlation finds are similar to [17] where number of participants was higher.

- Lines 632 and 649: "TEP's" should read "TEPs". The apostrophe should be used to indicate either possession or the omission of letters or numbers, but not to create plural forms of acronyms.

- Thank you this has been corrected.

- The spelling used is inconsistent, mixing British English and American English. I would advise using the former, correcting mistakes like "center" (should read "centre"), "analog" (should read "analogue"), "setup" (should read "set-up"), etc.

- This has been corrected.

- "Data" should be treated as a plural noun in scientific contexts (i.e. "data was" should read "data were"). Please correct this throughout the manuscript.

- This has been corrected.

References:
Tononi et al. (1998) Complexity and coherency: integrating information in the brain, Trends in Cognitive Sciences, Vol. 2, No. 12, 474-484.

___________________________________________________________________

**Reviewer #2:** In this paper, the authors addressed how EEG indices of differentiation and integration are altered by sub-anaesthetic levels of ketamine compared to normal wakefulness. They showed that perturbational complexity index (PCI), which assesses differentiation as well as integration, is not significantly different between sub-anaesthetic levels of ketamine and normal wakefulness. By contrast, Lempel Ziv complexity (LZc), amplitude coalition entropy (ACE), or synchrony coalition entropy (SCE), which assesses differentiation alone, is higher in sub-anaesthetic levels of ketamine compared to normal wakefulness. The authors concluded that these indices reflect distinct aspects of consciousness. Specifically, they proposed that PCI reflects the brain’s ability to sustain consciousness, whereas LZc, ACE, or SCE reflects the richness of conscious contents.

Overall this is a solid paper. However, there are places where the lack of methodological details has rendered the conclusions / interpretations unjustified. The paper will benefit from a minor revision that clarifies the following points:

Thank you for your comments on the manuscript. We hope our clarification to your comments suffice for your approval of the manuscript for publication.

1. Disentangle the effect of analysis from the effect of data: The perturbational complexity index (PCI) is calculated from TSM-evoked EEG activity. By contrast, the Lempel Ziv complexity (LZc), amplitude coalition entropy (ACE), or synchrony coalition entropy (SCE) is calculated from spontaneous EEG activity. Therefore, it is unclear whether the differences between these indices in their changes (or lack of changes) from normal wakefulness to sub-anaesthetic levels of ketamine are due to the data themselves being differently affected by sub-anaesthetic levels of ketamine, or are instead due to the analyses / indices being differently affected by sub-anaesthetic levels of ketamine. It will be nice if the authors can provide some standard analyses which can be applied to both the TMS-evoked EEG activity and the spontaneous EEG activity and assess whether the data themselves are differently affected by sub-anaesthetic levels of ketamine. Or if performing such analyses will be difficult, it will be nice if the authors can provide some discussion on this issue.

- It is correct that we cannot be certain of whether the differences in effect of the ketamine condition seen in PCI and signal diversity measures are due to changes in the data or differences in the analysis applied. We have assumed that the data the analyses are based on would be affected differently by the ketamine intervention but have not yet shown this. We include a figure in the supporting information showing examples of the raw data, as well as power spectral densities, from both conditions, to give the reader an idea of how the data themselves change. We believe that this addition will at the very least improve the quality of the paper, as a presentation of the raw data was indeed lacking in the original version of the manuscript. That said, one of the main reasons for applying analyses based on both perturbational vs observational data is that the observed changes in derived measures may be different. The perturbational data are assumed to be better suited for disentangling differences in the effective structure of the brain directly, while the results of analyses based on observational data are more prone to being confounded by non-causal correlations in the data. Although, we feel that investigating how the raw signals (spontaneous and evoked) change using more standard methods (e.g. spectral analyses or peak latency and amplitudes) might be interesting in itself, we feel that doing such analyses might take focus away from the main aims of the paper. Therefore, we keep this to a minimum here (only presenting the data in a figure in the supporting information), and include the findings in the discussion:
  - Spectral analyses show that power spectrum density differences between wake and ketamine change for spontaneous data with eyes open and eyes closed, as well as for perturbational data, with different frequency band changes for each condition (S4 Fig).
  - Although perturbational data show frequency band changes in wake versus ketamine data, PCI seems less sensitive to changes in content (e.g. induced by removing visual aspects from phenomenal experience) within a global physiological state (here: being fully awake).
  - **S4 Fig. Spectral characteristics of the data.** Here we show examples of processed data from within a single epoch in the awake (a) and ketamine (b) conditions, as well as mean power spectral densities (PSD) across participants for wakefulness and ketamine (c) and the within subject, pairwise mean difference between wake and ketamine PSDs (d). Each of these are shown for the recordings of spontaneous activity in the resting state with eyes open (column 1) and resting state with eyes closed (column 2), as well as in response to TMS stimulation with eyes open (third column). In (a) and (b), the thick line shows the activity of channel Cz in the single epoch, while the gray lines in the background shows the activity of the remaining channels in that same epoch. For column 3, the black dashed line shows the time of the TMS pulse. In (c), the PSD is shown between 0 and 45 Hz, for the wakefulness (black) and ketamine (red) conditions. The thick line indicates the mean across participants, while the shaded area shows the extent of 3 standard errors of the mean. For the third column, the PSD is calculated only for the data after the pulse (0-500ms). The thick blue line in (d) shows the mean pairwise difference in the PSD (within participants) while the shaded area again shows the extent of 3 standard errors of the mean. Here we see that the frequency bands changes in the ketamine versus wakefulness states differ between the data types: in the eyes open condition, differences appear in the beta and low gamma bands (~12-30Hz); in the eyes closed condition, differences appear in theta, alpha, and low gamma bands (~4-12 Hz and ~20-30 Hz); in the perturbed data, in the alpha and beta bands (~8-20 Hz).

2. Lack of experimental details: The authors proposed that the increase in Lempel Ziv complexity (LZc), amplitude coalition entropy (ACE), or synchrony coalition entropy (SCE) from normal wakefulness to sub-anaesthetic levels of ketamine reflects the enriched conscious contents induced by ketamine. As part of the argument, they show that Lempel Ziv complexity (LZc), amplitude coalition entropy (ACE), or synchrony coalition entropy (SCE) is higher under eye open condition compared to eye close condition. However, the authors did not explain whether the eye open data were collected in a fully dark room (which are often the case for visual experiments), or in a normal room. If the eye open data were collected in a fully dark room, it is possible that the visual experiences would be richer under the eye close condition compared to the eye open condition, as closing the eye may facilitate visual imagery. If indeed so, this would contradict the authors’ argument. On a related note, the author did not examine whether the impacts of experimental condition (eye close versus eye open) on Lempel Ziv complexity (LZc), amplitude coalition entropy (ACE), or synchrony coalition entropy (SCE), differs significantly between sub-anaesthetic levels of ketamine and normal wakefulness. If the visual experiences under sub-anaesthetic levels of ketamine are largely induced by top-down visual imagery, as opposed to bottom-up visual inputs from environment, having the eyes closed versus open would have weaker impacts here, compared to the impacts they produced during normal wakefulness.

- Thank you for your comment. All the data were collected in a slightly dimmed, but light room. This was done to minimize eye blink artefacts in the TMS-EEG data. The suggestion that closing eyes may facilitate visual imagery is an interesting one, but since the eyes-open condition in a light operating room, we do not have the data for this comparison. We have added a comment in the methods section indicating that eyes-open condition sas not done in the dark.
  - “The recordings for the eyes-open conditions were done with the lights in the operating theater slightly dimmed.”
- Regarding the second point, we did investigate the interaction effect between eyes open/closed and sub-anaesthetic ketamine and normal wakefulness on signal diversity values, as presented in the result section:
  - “For the spontaneous signal diversity measures (LZc, ACE and SCE), there was no statistically significant two-way interaction between condition (normal wakefulness and sub-anaesthetic ketamine) and participants having eyes open or closed (LZc: *F*(1,9) = 0.85, *p* = 0.38, ACE: *F*(1,9) = 1.44, *p* = 0.26, and SCE: *F*(1,9) = 1.27, *p* = 0.29).”

We interpret this to mean that the effect of eyes open/closed on signal diversity is not dependent on whether you are in a normal wakeful state or in a sub-anaesthetic ketamine state. We realize that this finding is not expressed in the discussion and have therefore included a sentence to clarify:

- “We found that for all spontaneous signal diversity measures, there was a significant decrease when participants had their eyes closed compared to when they had open eyes. This was independent of whether the participants were in a normal wakeful state or in the sub-anaesthetic ketamine state.”

We also added a clarification regarding this point in the methods section:

- “The analysis was designed to uncover effects of the conditions (normal vs ketamine and eyes open vs eyes closed) as well as any interaction effect between the conditions.”

**3.** Asymmetry in experimental condition: In this study, perturbational complexity index (PCI) / TMS-evoked EEG data were acquired under the experimental condition of eye open; by contrast, Lempel Ziv complexity (LZc), amplitude coalition entropy (ACE), or synchrony coalition entropy (SCE) / spontaneous EEG data were collected under the experimental condition of eye open as well as the experimental condition of eye close. It would benefit the readers if the authors could provide rationales behind such experimental design: namely, why there lacked the eye close condition for perturbational complexity index (PCI) / TMS-evoked EEG data.

- We did not include an eyes closed condition for PCI measurement because we did not want participants to be longer under sub-anaesthetic ketamine than necessary, and studies investigating closing the eyes have not found this to influence PCI results. This is stated in the discussion:
  - In contrast, having eyes closed does not seem to affect the TMS-EEG response [41, supplemental material] nor the PCI values during wakefulness [12, supplemental material].”
- However, we also added a description in the methods section, outlining this asymmetry in the experimental condition:
  - “Thereafter, participants were given an intravenous infusion of sub-anaesthetic doses of ketamine. When the participant reached a state in which they reported a noticeable effect of the drug, we measured the RMT again [27], and adjusted the TMS stimulation intensity to the same percentage of the RMT as was used during the wakeful state. We then delivered another 300 TMS pulses, while keeping the stimulation target the same as before ketamine administration. In addition, spontaneous EEG was recorded under the same conditions, once again asking the participants first to keep their eyes open and then to have their eyes closed. The TMS-EEG measurements were only done with eyes open to keep the time spent under the effect of ketamine short, as the required measurements take at least 10 minutes and the ethical approval only allowed one set of TMS-EEG measurements per condition (normal wakefulness and ketamine). ”
